# Diagnosis, treatment, and follow-up of patients with hypophosphatasia

**DOI:** 10.1007/s12020-024-04054-1

**Published:** 2024-12-12

**Authors:** Juan Guillermo Cárdenas-Aguilera, Vladimir González-López, Ana María Zarante-Bahamón, Juan Carlos Prieto-Rivera, Richard Baquero-Rodríguez, Kelly Rocío Chacón-Acevedo, Adriana Isabel Meza-Martínez, Ana Katherina Serrano-Gayubo, Adriana Medina-Orjuela, Jimena Adriana Cáceres-Mosquera, Gustavo Adolfo Guerrero-Tinoco, María Fernanda García-Rueda, Pilar Guarnizo-Zuccardi, Gilberto Herrera-Ortiz, Carolina Rojas-Barrera, Martha Isabel Carrascal-Guzmán, María Fernanda Reina-Ávila, Sletza Lissette Arguinzoniz-Valenzuela, Leticia Belmont-Martínez, Mariana del-Pino, Gisela Lorena Viterbo, Mariana Seijo, Joan Calzada-Hernández, Norma Elizabeth Guerra-Hernández, Oscar Héctor Brunetto

**Affiliations:** 1 Subred Integrada de Servicios de Salud Sur Occidente, Bogotá, Colombia; 2 Asociación Colegio Colombiano de Endocrinología Pediátrica, Bogotá, Colombia; 3 Hospital Universitario San Ignacio, Bogotá, Colombia; 4 Pontificia Universidad Javeriana, Facultad de Medicina, Bogotá, Colombia; 5 Universidad de Antioquia, Facultad de Medicina, Medellín, Colombia; 6 Grupo de Investigación Traslacional, Keralty, Bogotá, Colombia; 7 Instituto Roosevelt, Bogotá, Colombia; 8 Hospital San José, Bogotá, Colombia; 9 Clínica Infantil Santa Maria del Lago, Bogotá, Colombia; 10 Universidad de Cartagena, Bolívar, Colombia; 11 Fundación Cardioinfantil, Bogotá, Colombia; 12 Clínica Comfamiliar, Risaralda, Colombia; 13 Fundación Hospital de la Misericordia, Bogotá, Colombia; 14 Universidad del Valle, Cali, Colombia; 15 Centro Médico ABC, Ciudad de México, México; 16 Instituto Nacional De Pediatría, Ciudad de México, México; 17 Hospital de Pediatría Garrahan, Buenos Aires, Argentina; 18 Sociedad Latinoamericana de Endocrinología Pediátrica, Buenos Aires, Argentina; 19 Instituto de Inmunología, Genética y Metabolismo, Buenos Aires, Argentina; 20 Hospital Sant Joan de Déu, Barcelona, España; 21 Hospital General del Centro Médico Nacional La Raza, Ciudad de México, México; 22 Hospital de Niños Pedro de Elizalde, Buenos Aires, Argentina

**Keywords:** (DeCS): Hypophosphatasia, Alkaline phosphatase, Diagnosis, Treatment, Consensus

## Abstract

**Introduction:**

Hypophosphatasia is a rare inherited systemic metabolic disorder, with an estimated prevalence in the severe forms of the disease of 1/100.000–1/300.000, that affects the typical architecture of bone, leading to defective mineralization during growth and remodeling. It is characterized by loss-of-function variants in the *ALPL* gene, resulting in low activity of tissue-nonspecific alkaline phosphatase. In severe cases, it can be fatal.

**Objective:**

To generate recommendations on the diagnosis, treatment, and follow-up of patients with hypophosphatasia based on available evidence.

**Materials and methods:**

A search for evidence published between 2012 and 2024 was carried out in Medline and Embase. The search was expanded with information from various sources, including official sites of development groups, consensuses, technology evaluations, Google Scholar, clinical experts, and reference lists. The quality of the evidence was evaluated according to the type of document type. A modified Delphi consensus process was carried out with external experts, apart from the development group, it was established an 80% agreement threshold to define the final recommendations.

**Results:**

Sixty-one papers were found in the evidence search. The global quality of the evidence was low. In addition, a consensus was reached on 94 recommendations regarding diagnosis, treatment, and follow-up. Those recommendations were approved by external clinical experts from Colombia, Argentina, Spain, and Mexico.

**Conclusions:**

The recommendations proposed in this document are based on the evidence available to the date the search was carried out, and the judgment of clinical experts. The recommendations on diagnosis, treatment, and follow-up are expected to guide the daily clinical practice for patients with HPP.

## Introduction

Hypophosphatasia (HPP) is a rare, hereditary disease, with an estimated prevalence in the severe forms of 1/100.000–1/300.000, that mainly affects mainly the bone metabolism and its architecture [1]. HPP is caused by variants in the *ALPL* gene, which encodes tissue non-specific alkaline phosphatase (TNSALP). This results in a deficiency in the activity of this enzyme. TNSALP plays a crucial role in bone and tooth mineralization, regulating extracellular substrates such as inorganic pyrophosphate (PPi), pyridoxal-5-phosphate (PLP), and phosphoethanolamine (PEA) [1–6].

In patients with hypophosphatasia, the accumulation of PPi compromises normal bone architecture, leading to radiological changes like those seen in rickets and osteomalacia. These patients may also present with bone fragility and increased risk of angular bone disorders, fractures, and pseudofractures [6–9]. PLP is an essential component of pyridoxine (vitamin B6), which is a cofactor in the synthesis of neurotransmitters and allows the conversion of levodopa to dopamine, therefore facilitating the conversion of the excitatory neurotransmitter glutamate into the inhibitory neurotransmitter GABA; the accumulation of PLP in patients with severe neonatal forms of HPP causes an increased risk of seizures. These seizures are complex and difficult to handle but can be treated with pyridoxine [7, 9–11].

HPP exhibits significant genetic heterogeneity, with over 410 pathogenic variants of the *ALPL* gene reported. This genetic diversity accounts for the clinical variability and the specific manifestations observed in patients [7, 9, 12–15]. The characteristics and severity of HPP can vary widely, even within the same age group and among affected members of the same family [8, 14, 16–18]

HPP rises big challenges when it comes to understanding its genetic basis, clinical spectrum, diagnosis, management, and follow-up strategies [3, 13, 17, 19–21]. This consensus aims to provide recommendations on those topics, based on the most recent available scientific evidence and, in the absence of such evidence, the expertise of the developer group.

## Materials and methods

**Fig. 1:**
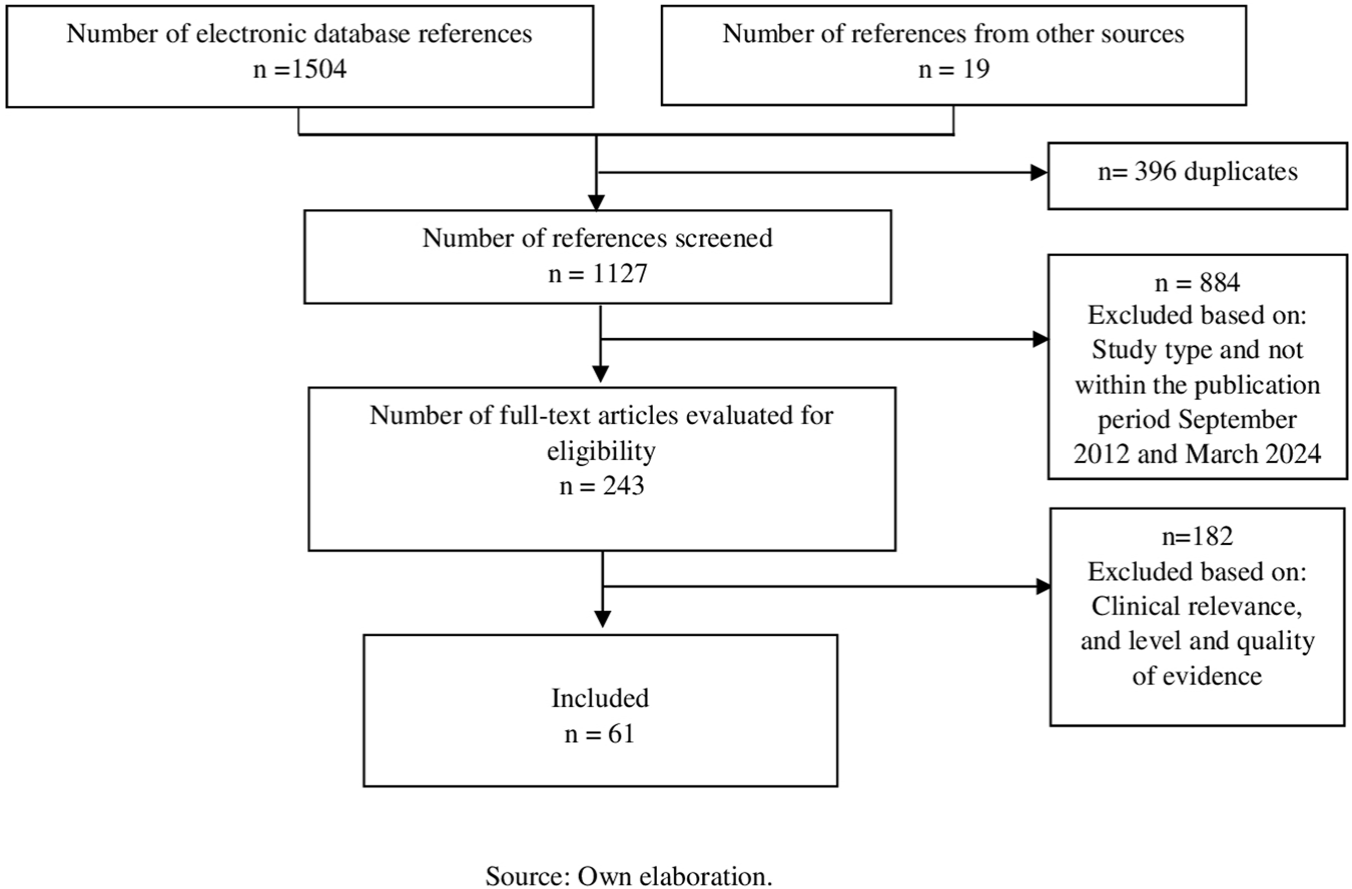
Flow chart – Prism. Source: Own elaboration

This evidence-based consensus was developed by a multidisciplinary task force comprising clinical experts in genetics, endocrinology, orthopedics, rheumatology, and nephrology, covering both pediatric and adult care. Additionally, methodologists experienced in evidence research and consensus development were recruited. Nine key questions were identified and answered based on systematic search for evidence in the Medline (Pubmed) and Embase databases (Fig. 1). This search was supplemented with gray literature from Google Scholar. Studies published in Spanish or English between September 2012 and February 2024 were included. Two reviewers (clinical-methodologist experts, independently selected the relevant information. The quality of the evidence was evaluated according to the document type; AGREE II was used for clinical practice guidelines (CPGs), ROBIS for systematic reviews (SRs), and the Joanna Briggs Institute tools for primary studies. Recommendations were discussed by the development group in virtual synchronous sessions, followed by asynchronous consultations with external clinical experts. An agreement threshold of at least 80% was established for each recommendation. The recommendations were graded by intensity (strong or weak/conditional) and by direction (in favor or against) [22].

## Results

Sixty-one documents were obtained from the search; defined as the body of evidence, to answer the questions proposed by the development group. Consensus was reached on 94 recommendations on diagnosis, treatment, and follow-up, which a group of external clinical experts from Colombia, Argentina, Spain, and Mexico, approved.

The overall quality of the evidence was low, primarily due to the studies design, which included case reports, case series, and narrative reviews. Some documents provided post hoc analyses, and the only clinical practice guideline (CPG) identified, had limitations in its methodological rigor and editorial independence. It is crucial to recognize that HPP is a rare disease, making studies with significant sample sizes uncommon and challenging to conduct.

The recommendations approved by the expert group, along with the evidence synthesis, are presented below.

### Question 1. What is the clinical presentation of patients with HPP?

#### Recommendations

1. In the pediatric population, it is recommended to suspect HPP in the presence of the following symptoms and signs (Table 1): short stature, angular and rotational deformities of the long bones, Chiari malformations, hydrosyringomyelia and craniosynostosis, wide fontanel or delayed closure, delayed motor development, gait disturbances, hypotonia, musculoskeletal pain, muscle weakness, limitation in mobility and premature loss of deciduous teeth (before the age of six years). (Strong in favor).

**Table 1:** Diagnostic criteria for HPP

Diagnostic criteria for HPP in children	Diagnostic criteria for HPP in adults
**Major**	**Major**
Pathogenic or likely pathogenic ALPL gene variant	
Elevation of natural substrates (measurement of plasma vitamin B6 requires stopping pyridoxine supplementation 1 week prior to measurement)	
Early nontraumatic loss of primary teeth	Atypical femoral fractures (pseudofractures)
Presence of rickets on radiographs	Recurrent metatarsal fractures
**Minor**	**Minor**
Short stature or linear growth failure over time	Poorly healing fractures
Delayed motor milestones	Chronic musculoskeletal pain
Craniosynostosis	Early atraumatic loss of teeth
Nephrocalcinosis	Chondrocalcinosis
B6 responsive seizures	Nephrocalcinosis

Source: adapted from [2]
2. In the adult population, it is recommended to suspect HPP in cases with the following symptoms or signs (Table 1): deformities described in childhood, in addition to osteomalacia, joint stiffness or limited mobility, osteoarthritis, fragility fractures, delayed osteosynthesis, pseudofractures, and atraumatic loss of teeth. (Strong in favor).
3. HPP should be considered in pediatric cases of seizures responsive to vitamin B6 or nephrocalcinosis. (Strong in favor).

#### Evidence

Six categories of the disease have been described based on the age of symptoms onset: lethal perinatal, benign perinatal, infantile, juvenile, adult, and odontohypophosphatasia [23, 24].

Lethal HPP is named so because death occurs in utero or the first weeks of life due to the absence of skeletal mineralization, pulmonary hypoplasia, rib fractures, or pyridoxine-responsive seizures that are non-responsive to usual anticonvulsant treatment [23, 25]. In benign perinatal HPP, limb shortening with bowing of the long bones is observed. This can be detected ultrasonographically in utero and tends to show spontaneous improvement in the last gestational trimester or at birth. Infantile HPP can become evident during the first six months of life and has a poor prognosis for survival in the first year of age if it is not properly treated [23, 24]. At least 50% of the mortality in patients with lethal or infantile form, are related with bone abnormalities in the ribcage that lead to respiratory failure. The earlier the signs of HPP appear, the more severe the disease's impact and burden. Without treatment, the prognosis for these patients is poor [25, 26].

In patients with juvenile HPP, symptoms appear after the first six months of life but before 18 years of age. It is characterized by bone hypomineralization, short stature, delayed psychomotor development, multiple fractures, gait disturbance, chronic bone pain, hypotonia, gastrointestinal problems, cavities, and teeth loss [5, 23]. Adult HPP is diagnosed when patient’s manifestations begin after the age of 18 years, main characteristics in those patients include: fractures (mainly in the lower extremities), osteomalacia, osteoporosis, delayed fracture healing, chondrocalcinosis, ectopic calcifications [27], and kidney compromise such as nephrocalcinosis and kidney stones [5, 23]. Patients with odontohypophosphatasia presents with premature loss of permanent or deciduous teeth with intact roots; a hallmark of dental involvement. It may also be associated with severe caries, periodontal disease, and alveolar bone loss without systemic involvement [23].

Clinically, HPP patients can be symptomatic or asymptomatic. Pain is a common symptom (with a frequency between 41 and 64.5%) [15, 28], along with muscle stiffness and weakness [23]. Bone manifestations are characterized by growth deceleration due to the impact on the endochondral ossification and mineralization process. At the skull level, craniosynostosis, plagiocephaly, and wide fontanelles can occur [29]. In long bones, metaphyseal involvement, diaphyses bowing, significant deformities, and bone fragility can be found (Table 2). Thoracic kyphoscoliosis of the spine is also seen [2, 27, 30, 31].

**Table 2:** Normal intercondylar and intermalleolar distances by age

Age	Intercondylar distances	Intermalleolar distances
6 months	0–5 cm	0–2 cm
12 months	0–4 cm	0–2 cm
>2 years	0–2 cm	<6 cm

Source: adapted and modified from [60].

Some patients may experience manifestations that begin in childhood, such as craniosynostosis, rickets, muscle weakness, and respiratory difficulty due to chest deformities, among others, and be diagnosed late, in adulthood [32, 33]. Enthesopathy, fractures, and pseudofractures (Looser-Milkman lines) are common in adults and are reported in 54% of cases [2, 28]. Other manifestations, such as respiratory and kidney symptoms and chronic pain, are common in more severe forms of the disease [14].

To establish the diagnosis of HPP in children and adults, it is necessary to evaluate within the context of major and minor clinical criteria, using a combination of either two major criteria or one major and two minor criteria [2].

### Question 2. Which biochemical studies are helpful to diagnose HPP?

#### Recommendations

1. It is recommended to measure Alkaline phosphatase (ALP) using the colorimetric method in all patients with clinical or radiographic suspicion of HPP, comparing the result with the reference values for age and sex (Table 3). (Strong in favor).

**Table 3:** Normal ALP values by age and sex

Age	Alkaline Phosphatase (IU/L)	
	Male	Female
0 to 14 days	90–273	90–273
15 days to < 1 year	134–518	134–518
1 year to < 10 years	156–369	156–369
10 years <13 years	141–460	141–460
13 years < 15 years	127–517	62–280
15 years < 17 years	89–365	54–128
17 years < 19 years	59–164	48–95
Adults	40–150	40–150

Source: adapted [61, 62]
2. At least two different ALP measurements are recommended in patients with suspected HPP, and both must be low for age and sex to confirm the diagnosis. (Strong support).
3. Bone-specific ALP measurement is not recommended for the diagnosis of HPP. (Strong against).
4. Other causes of low ALP should be ruled out (Table 4). These causes should be explored in an individualized manner, and relevant directed laboratories should be performed. (Strong in favor).

**Table 4:** Causes of low ALP

Persistenly low	
Metabolic bone disease	
*Hypophosphatasia*	*Cleidocranial dysplasia.*
*Msleny joint disease*	*Benign familial hypophosphatasemia*
**Temporary low**	
Endocrine conditions	
*Cushing syndrome*	*Hypothyroidism*
Metabolic bone disease	
*Osteogenesis imperfecta, type II*	*Adynamic bone disease*
Hematological conditions	
*Massive transfusion*	*Perniseous or profound Anemia*
*Multiple myeloma*	
Diet related	
*Milk-alkali syndrome*	*Starvation*
*Vitamin C deficency*	*Vitamin D intoxication*
*Zinc or magnesium deficency*	
Others	
*Wilson disease*	*Cardiac bypass surgery*
*Celiac disease*	*Hemochromatosis*
*Radioactive heavy metal intoxication*	*Use of inappropriate reference ranges*
Inadequate blood collection (Oxalates, EDTA)	
Drugs	
*Clofibrate*	*Denosumab*
*Biphosphonates*	*Tamoxifene*
*Glucocorticoids*	*Chemotherapy*

Source: adapted from [7, 10]
5. In any patient with suspected hypophosphatasia or bone deformities, it is recommended to evaluate phospho-calcium metabolism [measurement of calcium, phosphorus, magnesium, creatinine, creatinuria, calciuria (in isolated urine or 24-hour urine collection), phosphaturia, 25-hydroxyvitamin D (25-OH-D), parathyroid hormone (PTH)], due to the probable findings of hypercalcemia, hyperphosphatemia, hypermagnesemia, hypercalciuria related to HPP. Those are also useful to rule out chronic kidney disease (CKD) and make a differential diagnosis. These values must be interpreted according to the patient's age (Table 5). (Strong in favor).

**Table 5:** Normal calcium and phosphorus values by age

Age	Calcium (mg/dL)	Phosphorus (mg/dL)
0 to < 1 month	8.7–11	5.6–10.5
1 month to < 1 year	8.9–10.9	4.8–8.4
1 year to 3 years	8.9–10.5	4.3–6.8
4 to 6 years	8.9–10.2	4.1–5.9
7 to 9 years	8.9–10.2	4.1–5.9
10 to 12 years	8.9–10.2	4.1–5.9
13 to 15 years	8.8–10.1	3.2–5.5
16 to < 18 years	8.6–10.1	2.9–5.0
> 18 years	8.5–10.5	2.5–4.5

Source: adapted from [60–62]

#### Evidence

The diagnosis of HPP integrates clinical, biochemical, and radiological findings. It is characteristic in laboratories to find diminished ALP activity compared to reference values for age and sex in HPP [26, 34] (Table 3).

Other causes of low ALP exist and must be analyzed and ruled out in patients with suspected HPP [24, 35] (Table 4).

The presence of hyperphosphatemia, hypercalciuria, hypercalcemia, and hypermagnesemia [27] are common in HPP [15, 33]. However, some patients may have normal levels of serum calcium, phosphorus, PTH, and 25-OH-D [24, 29, 34].

An increase in non-tissue-specific ALP substrates TNSALP, such as PPi, PLP in blood [7, 26, 36], and PEA in urine, have also been described [14], although their availability is limited [37].

### Question 3. Which imaging studies are recommended in HPP patients?

#### Recommendations

1. Correlating the patient's age with the clinical, biochemical, and radiological findings suggestive of HPP is recommended. (Strong in favor).
2. For patients with a family history of HPP, prenatal ultrasonography by a radiologist experienced or trained in skeletal dysplasias or a specialist in perinatal medicine is recommended. This helps in the evaluation of the presence of characteristic signs of HPP (shortening, angulation, or bowing of long bones and the presence of osteochondral spurs), enabling early diagnosis of lethal perinatal and benign perinatal HPP. (Strong in favor).
3. When bone alterations compatible with HPP (shortening or angulation of long bones or presence of osteochondral spurs) are detected in routine prenatal ultrasounds, referral to a perinatal specialist for confirmation and management of those findings is recommended. These manifestations could indicate early diagnosis of lethal perinatal and benign perinatal HPP. (Expert opinion).
4. Identifying characteristic signs of HPP in radiographic studies is recommended, such as irregular bones, widened sutures, brachycephaly, craniosynostosis, hammered copper sign, rachitic changes in the costal arches and costochondral junctions, widened metaphyses, poorly ossified epiphyses, and arched long bones. (Strong in favor).
5. For neonates suspected of HPP, a complete series of bones radiographs, including the skull, axial, and appendicular skeleton is recommended for diagnostic purposes. This helps identify suggestive signs of ossification alteration or bone mineralization, metaphyseal tongue shaped radiolucencies, angular deformities, and pathological fractures. (Strong in favor).
6. In infants suspected of HPP, performing a complete series of long bones radiographs, including the skull, axial, and appendicular skeleton, is recommended for diagnostic purposes to identify suggestive characteristics of HPP. (Strong in favor).
7. In radiographic studies of adults, recognizing signs suggestive of osteomalacia, enthesopathies, ectopic calcifications, deformities, widened metaphyses, fractures, and pseudofractures (usually in the lateral cortex of the subtrochanteric region of the femur), is recommended. (Weak in favor).
8. For adults diagnosed with HPP, biennial or annual renal ultrasonography is recommended to monitor for nephrocalcinosis and kidney stones. (Strong in favor).
9. For the pediatric population, renal ultrasonography is suggested at the time of diagnosis and every 3 to 6 months, depending on the patient's clinical characteristics. (Weak in favor).
10. Routine whole-body MRI for diagnostic purposes is not recommended in patients suspected of HPP. (Strong against).
11. Bone mineral density measured by dual-energy x-ray absorptiometry (DXA) is not recommended for diagnostic purposes of HPP. (Strong against).

#### Evidence

Imaging is considered a cornerstone for the diagnosis and follow-up of patients with HPP. Main radiological suggestive findings include: altered mineralization, metaphyseal tongues (radiolucencies), deformities, fractures, and pseudofractures [26, 36]. Table 6 shows the main radiological findings of HPP by age group, and Table 7 shows differential diagnoses by subgroup of disease classification [38].

**Table 6:** Clinical characteristics, complications and radiological findings of HPP by age group

Perinatal period and Neonatal period	
Radiological findings	Long bones: shortening, arching, angulation.
	Small/narrow chest (chest size is smaller than abdominal circumference)
	Osteochondral spicules (Bowler's spicule)
	Metaphyseal irregularities – radiolucent (“widening” or “tongues”)
	Ribs: Short and excavatum^a^ Thin^b^
	Poor/absent ossification of bones: tubular bones, domed skull, ribs, vertebrae
	Hypoechoic skull with increased nuchal sonolucence
	Large sutures and fontanelles^b^
	Polyhydramnios
Clinical signs and Complications	Neurological: seizures non-responsive to anticonvulsant treatment
	Bones: fractures, bone deformities, craniosynostosis, Limbs shortening
	Pulmonary: respiratory difficulty and failure, respiratory infections, pulmonary hypoplasia.
	Kidneys: nephrocalcinosis
	Calcium metabolism: hypercalcemia and hypercalciuria
	Thoracic deformity: knobs of bone at the costochondral joints, rib fractures
**Childhood**	
Radiological findings	Fractures
	Limbs bones deformities/shortening
	Craniosynostosis
	cranial radiograph showing cooper-beaten pattern
Clinical signs and Complications	Musculoskeletal: Bone pain, myopathy/hypotonia, osteoarthralgia, myalgia, muscle weakness, gait alterations, physical disability, functional limitation, gait abnormality/delay
	Neurological: seizures non-responsive to anticonvulsant treatment, delayed motor development, Chiari malformation, hearing loss, neuropathy
	Growth retardation, short stature
	Dental: Early loss of deciduous teeth, periodontal disease
	Hyperphosphatemia, hypercalcemia and hypercalciuria
	Kidneys: nephrolithiasis, nephrocalcinosis
**Adulthood**	
Radiological findings	Fractures
	Delayed fracture healing / Pseudarthrosis
	Osteomalacia
	Osteoporosis
	Limbs bones deformities/shortening
Clinical signs and Complications	Neurological: hearing loss, neuropathy
	Dental: periodontal disease, loss of permanent teeth
	Musculoskeletal: Recurrent fractures, bone deformities, bone pain, osteomalacia, osteoporosis, osteoarthralgia, myalgia, muscle weakness, pseudogout, gait alterations, physical disability, functional limitation, abnormal gait
	Kidneys: nephrolithiasis, nephrocalcinosis
	Hyperphosphatemia, hypercalcemia and hypercalciuria
	Growth: final short stature

^a^Second trimestre (betwen the 13th and 27th gestational weeks)

^b^Term Newborn

Source: adapted and modified from [26, 38, 41]

**Table 7:** HPP differential diagnosis based on radiological findings

Stage	Differential diagnosis
Antenatal Ultrasonography Fetal CT	Imperfect osteogenesis Campomelic dysplasia Caffey disease or Caffey-Silverman syndrome Kyphomelic dysplasia Chondrodysplasia with mineralization defect Cleidocranial dysplasia Stuve-Wiedemann syndrome
Pediatric	Rickets Metaphyseal dysplasia Imperfect osteogenesis Idiopathic osteoporosis Cole-Carpenter syndrome Hadju-Cheney syndrome Mucolipidosis type 2 Chronic recurrent osteomyelitis Multifocal osteomyelitis
Adult	Osteoporosis Imperfect osteogénesis Chondrocalcinosis X-linked hypophosphatemia Osteomalacia Early osteoarthritis

Source: adapted from [41]

Fetal ultrasound (USG) allows the identification of mineralization defects, shortening, bowing, or spurs of long bones [9]; its early performance has been recommended to achieve an early diagnosis and improve outcomes [26]. Renal ultrasonography helps monitor nephrocalcinosis and kidney stones in the adult population [15, 23, 39]. Although the diagnostic usefulness of renal ultrasound in pediatric patients is not clear, a frequency of nephrocalcinosis of around 38% has been found (50% in perinatal HPP, 76% in childhood HPP, and 17% in pediatric HPP) [40]. Some authors suggest performing USG to detect nephrocalcinosis only in cases of hypercalcemia and hypercalciuria [41].

Long bone radiographs allow us to identify ectopic calcifications, bone mineralization alterations, angular deformities, osteomalacia, and widened metaphyses [5, 27]. Skull X-rays can show increased convolutional marks, suggesting fusion and early closure of sutures (hammered copper sign). Signs of hypomineralization with apparently very opened sutures and wide fontanelles can also be observed in Skull X-rays [23, 39].

Intrauterine 3D helical computed tomography (CT) is a technique that can provide detailed bone images, improving diagnostic accuracy. However, its use is indicated from 28 or 30 weeks of gestation, and it is used only in specific cases with careful evaluation of risks and potential benefits [8].

Magnetic resonance imaging (MRI) is considered a sensitive but nonspecific tool for detecting early changes in bone structure and inflammatory signs of the musculoskeletal system, regardless of age [39]. However, it has not been routinely recommended for the diagnosis of HPP.

Bone mineral density (BMD) can be normal [42, 43], but also, in the course of the disease, it can be low, and associated with hypomineralization in severe cases [14, 41]. Although BMD is studied in patients with bone metabolism alterations, it is not a diagnostic tool in patients with HPP [44].

It is necessary to rule out differential diagnoses based on imaging findings (Tables 6 and 7) to avoid misdiagnosis of osteopenia or primary osteoporosis, which can lead to inappropriate treatment with bisphosphonates [23, 41].

### Question 4. In which patients is genetic testing indicated to confirm HPP?

#### Recommendations

1. Next-generation sequencing (NGS) and copy number variation (CNV) of the *ALPL* gene are recommended for all patients with clinical, biochemical, and radiological findings compatible with HPP diagnosis. (Strong in favor).
2. Genetic testing is essential for evaluating inheritance mechanisms, provide genetic counseling about the risk of recurrence, reproductive alternatives, and assessing at-risk family members. (Strong in favor).
3. For patients with negative NGS and CNV results of the *ALPL* gene, it is recommended to continue studying *ALPL* deletions and duplications using the MLPA (Multiplex Ligation-dependent probe amplification) technique. (Strong in favor).
4. It is proposed to follow the algorithm developed by the group for the genetic confirmation of the diagnosis of HPP (Illustration 1). (Strong in favor).
5. Genetic studies should be requested and interpreted by a geneticist or a specialist in HPP. (Expert opinion).

Illustration 1. Diagnostic algorithm.
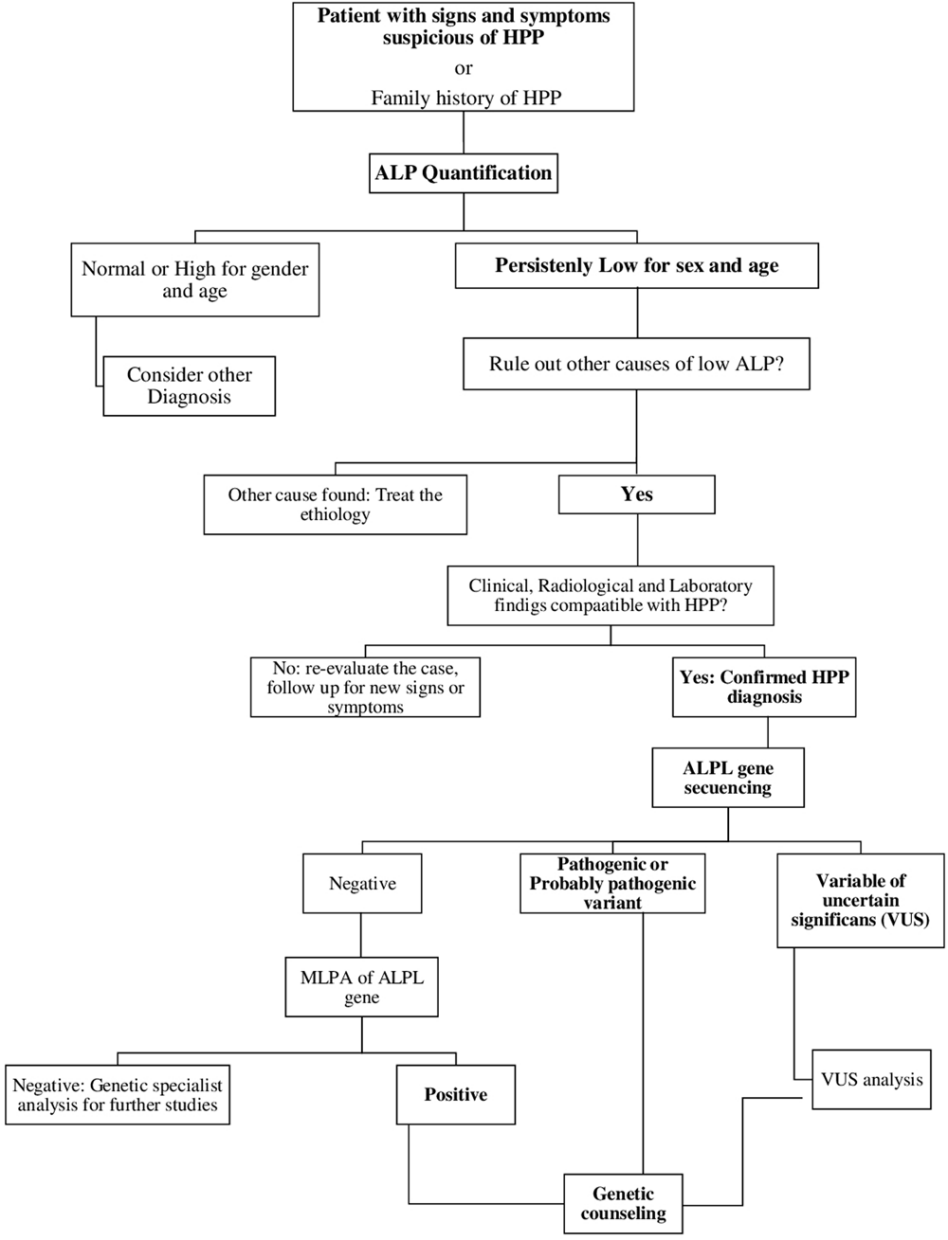


*two or more low ALP measurements adjusted for age and sex

** It is recommended to perform it, if available.

Authors elaboration

#### Evidence

Although HPP diagnosis is clinical and biochemical, genetic studies are essential to confirm certain diagnoses, rule out or evaluate differential diagnoses [24], and provide adequate genetic counseling based on the inheritance mechanism [23, 26, 45]. The recommended technique to find pathogenic or probably pathogenic variants in the *ALPL* gene is a next-generation sequencing (NGS) with copy number variation (CNV) analysis, which detects approximately 95% of variants [19]. The remaining 5% may be due to deletions or duplications in the gene or variants in regions not covered by this technology [46].

In 95% of patients with severe HPP (perinatal and infantile), variants of the *ALPL* gene can be identified [47, 48]. The American College of Medical Genetics and Genomics (ACMG) classification system is currently used to evaluate the pathogenicity of variants [34, 41, 49]. The variation status (biallelic or monoallelic) is only one aspect of the genotype-phenotype correlation. Homozygosity is often associated with more severe and earlier forms of the disease, which also relate to the type of variant [19]. However, the phenotypic spectrum of HPP is highly variable and depends on the age of onset, the inheritance pattern, and the type of gene variant [7].

Genetic studies are also justified in patients with inconclusive clinical, biochemical, or radiological findings of prenatal HPP, especially in women with a history of children with the disease [25]. The potential usefulness of these studies has also been described in cases where enzyme replacement therapy (ERT) is considered as an alternative treatment [2, 14]. Molecular confirmation of HPP allows us to elucidate the disease's inheritance pattern within a family [2, 14]. Therefore, family screening is helpful and sometimes necessary since some heterozygous individuals may express a mild form of the disease [45].

All patients should receive genetic counseling regarding inheritance pattern, the probability of recurrence in offspring, and reproduction options and alternatives [23, 26, 45].

### Question 5. Which are the complications in a patient with HPP?

#### Recommendations

1. In patients with HPP, it is recommended to evaluate possible complications of the musculoskeletal, respiratory, central nervous and renal systems that may affect the patient's quality of life. (Strong in favor).
2. Mild to moderate-intensity physical activity under the supervision and guidance of a physical therapist is recommended. (Weak in favor).
3. Identifying neurological manifestations such as seizures and treating them promptly with pyridoxine and/or other anticonvulsants, alongside consultation with a pediatric neurologist for study and follow-up, is recommended. (Strong in favor).
4. Evaluating the emergence of CKD, nephrocalcinosis, and nephrolithiasis in all patients is recommended. (Strong in favor).
5. Actively searching for metabolic alterations such as hypercalcemia, hypercalciuria, hyperphosphatemia, and hypermagnesemia is recommended. (Strong in favor).
6. Evaluating for the presence of craniosynostosis and, if found, looking for signs of intracranial hypertension or associated complications is recommended. (Strong in favor).
7. Dental follow-up is recommended to detect periodontal disease and early teeth loss and provide treatment and prosthetic rehabilitation. (Strong support).

#### Evidence

There is a wide variety of complications associated with HPP (Table 6), which depend on the age of presentation and can lead to alterations in functionality and quality of life (Table 6) [9, 26, 27, 50, 51].

### Question 6. What are the goals of treat a patient with enzyme replacement therapy (ERT)?

#### Recommendations

1. Establishing specific targets at the beginning of enzyme replacement therapy (ERT) is recommended, tailored to each patient’s HPP presentation, to impact functionality, quality of life, pain reduction, mobility improvement, and oral health. (Strong in favor).
2. Normalizing ALP levels is not recommended as a therapeutic objective of ERT. (Strong against).
3. In patients with perinatal and infantile HPP, emphasizing ERT treatment objectives on improving survival, recovering ventilatory status, controlling seizures, adequate growth, and achieving developmental milestones is recommended. (Strong in favor).
4. Setting goals at the beginning of ERT in patients with juvenile HPP to improve growth and final height, mobility, neurological development, and bone mineralization is recommended. (Strong support).
5. In adult patients with HPP, directing ERT towards ameliorating pain, improving functional status, improving gait pattern, consolidating fractures and pseudofractures, and lessening their occurrence is suggested. (Weak in favor).

#### Evidence

ERT goals are primarily oriented toward clinical rather than biochemical outcomes, so therapy to increase ALP levels is not recommended [13, 19]. With therapy, improvements in survival rate and overall prognosis are expected [26], alongside positive changes in the radiological ricket score (RSS Tacher), increases in height z-scores [9], enhancement in muscle function according to the 6-minute walk test [26], and delays in the need for orthopedic surgery until growth completion [9]. Table 8 summarizes the treatment objectives by type of HPP.

**Table 8:** Treatment objectives in patients with HPP

Perinatal/infantile	Juvenile	Adults
Survival	Improved mobility	Patient with fractures: • Improvement of fracture consolidation • Decrease in the frequency of fractures • Reduction in the number of fractures
Improvement in ventilatory pattern	Improvement of musculoskeletal disorders	
Improvement of skeletal abnormalities	Radiological improvement	
Kidney failure prevention	Improvement in growth and development	
Improvement in growth and development	Prevention of nephrocalcinosis	Patients with and without fractures: • Functional status improvement • Strength and endurance improvement • Gait pattern improvement • Fatigue decrease • Reduction of pain • Bone quality improvement
Correction of craniosynostosis	Pain improvement	
Seizure control	Maintenance of adequate oral health	
Pain improvement	Quality of life improvement	
Maintenance of adequate oral health		
Quality of life improvement		Maintenance of adequate oral health
		Quality of life improvement

Source: adapted from [19]

### Question 7. When should patients with HPP start enzyme replacement therapy?

#### Recommendations

Treatment Indications:

1. Patients should be individually evaluated to determine whether ERT is appropriate and, if so, for how long. This could be done by a risk-benefit analysis of the therapy. (Strong in favor).
2. ERT should be started immediately in patients with poor-prognosis forms of HPP, such as severe perinatal and childhood forms. This should be done as soon as a diagnosis is made to improve vital prognosis. (Strong in favor).
3. ERT is recommended in patients with HPP forms with osteoarticular alterations and muscle weakness, even if they have a favorable prognosis. This indication aims to improve the symptoms mentioned above. (Weak in favor).
4. ERT is not recommended for cases of odontohypophosphatasia. (Strong against).
5. In adults with HPP and osteomalacia-associated complications, pseudofractures, intractable musculoskeletal pain requiring or unresponsive to opioids (spondyloarthropathies, sacroiliitis, enthesopathy, chondrocalcinosis, among others), fragility fractures, delayed consolidation, nonunion of fractures, disabling functional deterioration, failure or loosening of the osteosynthesis material, persistent instability or peri-implant fracture, initiating ERT is recommended. (Weak in favor).
6. Low bone mineral density measured by bone densitometry should not be used as a criterion to initiate ERT, nor is it useful for ERT monitoring. (Strong against).

Asfotase alfa ERT, length of treatment.

1. An individualized approach should be considered for Asfotase alfa ERT maintenance, primarily based on therapeutic objectives compliance. Currently, there are no criteria for therapy suspension in effective cases. (Strong in favor).

Future research recommendation:

1. Long-term follow-up studies are recommended to determine the effectiveness, safety, and length of long-term therapy, especially in cases of poor-prognosis HPP. (Expert opinion).

#### Asfotase alfa ERT Suspension

1. Therapy should be stopped in cases of poor compliance or removal by the patient or caregiver, despite providing information about the risks and benefits of therapy. (Strong in favor).
2. Therapy should be discontinued if treatment objectives are not reached after evaluating anti-Asfotase alfa antibodies, if available. (Expert opinion).

#### Therapeutic scheme of enzyme replacement therapy with Asfotase alfa

1. The recommended dose of Asfotase alfa is 6 mg/kg/week, administered according to the manufacturer's recommendations. (Strong in favor).
2. Patients and their families should be informed about the benefits and risks of available treatments. (Strong in favor).
3. HPP treatment should be holistic and conducted by an interdisciplinary team according to the patient's needs and context availability. This team could include experts in genetics, pediatrics, endocrinology, nephrology, palliative care, psychiatry, psychology, orthopedics, neonatology, physical medicine and rehabilitation, rheumatology, pulmonology, perinatology, radiology, internal medicine, neurology, nutrition, occupational and physical therapy, all ideally with bone diseases expertise. (Weak in favor).
4. Patients with severe neonatal forms should be admitted to an intensive care unit and evaluated for mechanical ventilation needs. (Strong in favor).
5. Musculoskeletal pain should be treated with pharmacological analgesic treatment in intermittent or continuous regimens, based upon the severity of the symptom. (Strong in favor).
6. Kidney function should be monitored when using non-steroidal anti-inflammatory drugs for a prolonged time. (Expert opinion).
7. Vitamin D treatment is recommended in patients with 25-hydroxyvitamin D values below 20 ng/ml, avoiding vitamin D toxicity. (Weak in favor).
8. Nutritional support is recommended for patients with childhood-onset HPP. (Strong in favor).
9. In the event of hypercalcemia, hyperphosphatemia, and/or nephrocalcinosis, restricting calcium, sodium, and phosphorus in oral and parenteral nutrition is recommended. (Strong in favor).
10. For hyperphosphatemia, treatment with non-calcium phosphorus binders is suggested to avoid the risk of hypercalcemia. (Strong in favor).
11. In cases of hypercalcemia, stopping vitamin D treatment and encouraging oral or parenteral hydration is recommended, considering the patient's status calcitonin and/or ERT could be necessary and should be used if available. (Strong in favor).
12. For infants with hypercalcemia, using low-calcium infant formula (<30 mg/100 g) is suggested. (Expert opinion).
13. Bisphosphonates are not recommended in HPP patients with hypercalcemia. (Strong against).
14. Routine administration of steroids for hypercalcemia treatment in HPP is not recommended but may be considered in specific cases. (Weak against).
15. Renal replacement therapy is suggested for patients with HPP and severe refractory hypercalcemia. (Expert opinion).
16. Neurosurgical management is recommended for patients with symptomatic craniosynostosis and intracranial hypertension or Chiari malformation. (Weak in favor).
17. Physiotherapy and supervised low-impact exercises are recommended to strengthen the muscle-bone unit, tailored to age, weight, and functional capacity. (Weak in favor.)
18. Orthopedists; preferably with experience in these pathologies, should recommend individualized management for patients with fractures, pseudofractures, stress fractures and the monitoring and management of lower extremity deformities and scoliosis. (Expert opinion).
19. Teriparatide should be restricted to adult patients when ERT is unavailable and used under the supervision of an expert. (Expert opinion).

#### Evidence

Patients with HPP must be treated and monitored by specialists in bone metabolism [16]. Patients and their families should receive a clear and comprehensive explanation of the disease and treatment alternatives; detailing the potential benefits and risks [9].

ERT has been proposed as an early treatment for patients with severe perinatal and childhood presentations [37]. It has also been proposed in better prognosis HPP patients. However, its indication is based on the presence of bone and muscle symptoms [26] or a significant quality of life burden [43]. In high growth velocity phases, alternative ERT regimes can be considered, such as lower doses and short treatment cycles [9].

Regulatory entities in the European Community [52], Canada [24], and Colombia (INVIMA 2020M-0019887) have approved ERT primarily for bone manifestations in pediatric-onset HPP patients. In the United States, the indication has expanded to benign perinatal, infantile, and juvenile-onset HPP [53]. However, some authors highlight the lack of evidence supporting its use in benign perinatal HPP [26].

There is no conclusive evidence on the effectiveness and safety of Asfotase alfa ERT in patients with odontohypophosphatasia [54] nor is there consensus on the benefit of ERT in the adult population [9]. However, some authors have defined therapy criteria for adults, including osteomalacia and associated complications, pseudofractures, intractable musculoskeletal pain requiring or unresponsive to opioids, presence of chondrocalcinosis with intractable pain, major fractures secondary to osteoporosis, delayed or incomplete fracture healing, and significant functional impairment with gait and mobility problems [24, 26, 55, 56].

ERT effectiveness should be evaluated by tracking symptoms and bone signs on images, laboratory parameters, respiratory and muscle function, quality of life, and dental follow-up [26]. Bone densitometry is discouraged for determining whether to begin ERT or for therapy follow-up [56].

Considerations for the administration of ERT are not the objective of this consensus, the authors suggest to follow the manufacturer's recommendations.

The recommended ERT dose in HPP patients is 6 mg/kg/week, administered according to different schedules [9]. In severe cases unresponsive to standard dosing, an increased dose up to 9 mg/kg/week, divided into 3 mg/kg/dose three times per week, is suggested [25]. Studies on ERT effectiveness report follow-up periods of up to seven years, limiting the generalization of results to longer periods.

ERT suspension criteria include patient or caregiver desire after being informed of the therapy’s risk and benefits, cases of lipodystrophy [24, 53], and adverse events.

Patients may require additional medical treatment beyond ERT. Table 8 presents the medical treatment objectives by type of HPP, and Table 9 presents the medical management.

**Table 9:** Considerations for prescribing Asfotase alfa in HPP

Indication	
Treatment of patients with perinatal/infantile or juvenile HPP.	
Dosage and availability	
Injection route	Subcutaneous.
Injection site	Alternate the injection site (between the abdominal area, thigh, and deltoid), to avoid the risk of lipodystrophy. Inject at a 45- or 90-degree angle (45-degree angle for low fat patients). Do not give injections to hot, red, inflamed, thickened, hardened or swollen areas.
Dose	In HPP of perinatal/childhood onset: 2 mg/kg three times a week or 1 mg/kg six times a week; the dose may be increased due to lack of efficacy up to 3 mg/kg three times a week.
	In juvenile-onset HPP: 2 mg/kg three times a week or 1 mg/kg six times a week.
	In cases of severe HPP with lack of response to ERT, it is suggested to increase the dose to 9 mg/kg/week, administered as 3 mg/kg/dose, 3 times a week.
	Divide the volume larger than 1 ml into 2 syringes for 2 injections at separate sites.
	Single-use vials in boxes of 1 or 12 vials with the following concentrations: 18 mg/0.45 ml; 28mg/0.7ml; 40 mg/ml; and 80 mg/0.8 ml.

Source: adapted from [9, 19]

Musculoskeletal pain can be managed with analgesics, either intermittently or continuously, depending on symptoms severity [9, 27].

Vitamin D treatment is essential for patients with 25-hydroxyvitamin D levels below 20 ng/ml, with follow-up to avoid toxicity [5, 9]. In cases of hypercalcemia, vitamin D replacement must be stopped [26, 37], and oral or parenteral hydration, alongside calcitonin, and/or ERT [26] should be initiated if available.

Early-onset nutritional support, including enteral nutrition with gastric or jejunal tube, may be required in some cases, ensuring caloric intake. Mild or moderate-intensity physical therapy and exercise are recommended to maintain physical capacity [9].

Bisphosphonates are not recommended due to their inability to be metabolized by patients with HPP [5, 26, 27]. Non-calcium phosphorus binders may be considered in cases of hyperphosphatemia [9]. Renal replacement therapies may be necessary for hypercalcemia treatment in HPP patients with CKD [26].

Case reports suggest teriparatide aids in fracture healing in adults with HPP, but its use is approved for adults only and should be limited to experienced specialists, with careful monitoring of bone markers, and considered only when ERT is unavailable [5, 9].

Anti-sclerostin monoclonal antibodies have shown promise in increasing bone formation and a reduction in bone resorption, but their use in HPP adult patients should be carefully considered due to significant adverse effects [5].

For symptomatic craniosynostosis, due to intracranial hypertension or Chiari malformation, neurosurgical management and follow-up are recommended [9].

Dental prostheses are proposed for pediatric patients with premature teeth loss to promote adequate maxillary growth, normal eruption of permanent teeth, language development, and socialization [9].

### Question 8. What are the risks of ERT treatment?

#### Recommendations

1. Healthcare professionals, caregivers, and patients should have adequate knowledge and prior training in ERT administration. (Strong in favor).
2. Caregivers and patients should be educated about the signs and symptoms of hypersensitivity reactions and anaphylaxis related to ERT treatment (Table 10) and the need to report these to health professionals if any of those happened. (Strong in favor).

**Table 10:** ERT Adverse events and management

Adverse event	Characteristics	recommendations
Allergic reactions	Anaphylaxis: respiratory distress, nausea, angioedema, rash Hypersensitivity reaction: Fever, vomiting, skin rash, headache, irritability, chills, general or localized pruritus, oral hypoesthesia	• Educate about associated signs and symptoms of anaphylaxis and hypersensitivity. • Defer the application of asfotase alfa if you develop fever or undergo any procedure under anesthesia. • Defer the application of asfotase alfa if any type of vaccination is applied 24 h prior. • In the event of anaphylaxis or severe hypersensitivity, interrupt the application of asfotase alfa and initiate adjuvant treatment. • Make a record and inform the treating physician about the associated adverse event. • Consider the risks and benefits of restarting asfotase alfa after a severe reaction.
Local reactions at the injection site	It includes local edema, skin erythema, changes in skin pigmentation, pruritus, pain, papule, nodule, and dermal atrophy.	• Monitor and document local reactions with each administration of asfotase alfa. • Reinforce the application technique and rotation of the application site of asfotase alfa at each visit. • Record and inform the treating physician of local events associated with the application of the drug. • Refer to dermatology for evaluation of changes in pigmentation or signs of dermal atrophy.
Lipodystrophy	Lipoatrophy and/or lipohypertrophy at the sites of asfotase alfa application	• Evaluate the condition of the skin and dermal quality with each application of asfotase alfa. • Record and report associated local dermal events to the treating physician. • Rotate the application site in each of the asfotase alfa injection sessions.

Source: adapted from [19]
3. Health professionals administering ERT should report any adverse events appropriately. (Expert opinion).
4. If hypersensitivity reactions occur, such as an allergy or anaphylaxis reaction, stopping ERT and evaluating specific medical treatments is recommended. (Strong in favor).
5. Restarting ERT in a patient who previously experienced anaphylaxis should involve an individualized risk-benefits analysis and strict monitoring during application. (Expert opinion).
6. Premedication with antihistamines and corticosteroids is recommended before restarting ERT in patients with a history of anaphylaxis or severe allergic reactions (Table 11). (Strong in favor).

**Table 11:** Management and desensitization of Asfotase alfa-associated anaphylaxis

Event	Treatment
Anaphylaxis associated with the application of monoclonal antibody	• Discontinue the administration of Asfotase alfa. • Monitor vital signs • Administer epinephrine • Administer antihistamine (diphenhydramine, hydroxyzine or chlorphenamine). • Administer intravenous corticosteroids. • Manage hypotension with intravenous fluids. • Administer B agonist (albuterol or salbuterol) via inhaler or nebulization. • Administer oxygen through nasal cannula or mask. • Initiate advanced cardiopulmonary resuscitation if necessary.
Reboot and desensitization of asfotase alfa following an anaphylactic event	• Start with premedication: Administer 1 dose of antihistamine (diphenhydramine, hydroxyzine or chlorferinamine) plus 1 dose of Acetaminophen or Ibuprofen. • Divide the total dose of Asfotase Alfa over 2 days with application of 50% of the dose on the first day and 50% on the second day. • Fragment the total daily dose into 4 doses and apply asfotase alfa, as follows:

	Dose and application time after premedication
**Day 1**	1- 30 min
	2- 60 min
	3- 90 min
	4- 120 min
**Day 2**	5- 0 min
	6- 30 min
	7- 60 min
	8- 90 min

Source: adapted from [19, 63, 64]
7. ERT administration sites should be rotated. (Strong in favor).
8. A detailed record of ERT application date and areas, along with any reactions at the injection sites, should be maintained. (Strong in favor).

#### Evidence

ERT administration in patients with perinatal, infantile, or pediatric-onset HPP has been associated with mild to moderate adverse reactions at the injection sites. 68 to 73% of cases are usually resolved within a week [19, 53]. Adverse reactions include erythema, rash, loss of pigmentation, pruritus, pain, papular or nodular lesions, hematomas, and lipodystrophy at the injection site [9, 19, 26]. Localized lipodystrophy, including lipohypertrophy and lipoatrophy at injection sites, can be prevented by rotating injection sites between the deltoid, abdomen, and thigh, and by reducing the number of administrations from six to three times per week [19, 26, 53].

Doctors must ensure that patients, parents, and caregivers are properly informed about the injection technique before letting them to administer the therapy on their own [26].

Other adverse events such as ectopic calcifications (14%), ophthalmic calcifications (conjunctiva or cornea), and nephrocalcinosis (14%) require biochemical and ophthalmological follow-up. Hypersensitivity reactions, including fever, headache, irritability, nausea, periorbital edema, and anaphylaxis have been reported in 12% of patients [19, 53]. In the event of a hypersensitive reaction, ERT should be stopped immediately, and symptoms should be treated. Before beginning ERT, a complete ophthalmological evaluation and renal ultrasonography are recommended to evaluate the presence or absence of calcifications [19]. Tables 10 and 11 summarize the therapy's adverse events and their proposed management based on the reviewed evidence.

### Question 9. How should clinical and paraclinical follow-up of HPP patients be done?

#### Recommendations

1. Periodic follow-up, including clinical, biochemical, and radiographic assessments, is recommended to evaluate disease progression, identify complications, and assess ERT effectiveness (Table 12). (Strong in favor).

**Table 12:** Follow-up schedule for patients on ERT by HPP type

	Start	2 weeks	1 month	3 months	6 months	9 months	12 months	every 3 months	C/6 months	Annual	C/2 years	As required
Alkaline phosphatase												
Perinatal/infant	X											X
Pediatric	X											X
Adult	X											X
Calcium												
Perinatal/infant	X		X	X	X		X			X		
Pediatric	X			X						X		
Adult	X			X						X		
PTH	X											X
Vitamin D 25 OH												
Perinatal/infant	X		X	X	X		X			X		
Pediatric	X			X	X					X		
Adult	X			X	X					X		
Phosphorus												
Perinatal/infant	X		X	X	X		X			X		
Pediatric	X			X						X		
Adult	X			X						X		
Routine tests (CBC, liver function, electrolytes)												
Perinatal/infant	X			X	X	X	X			X		
Pediatric	X				X					X		
Adult	X				X					X		
Kidney function (BUN, Creatinine)												
Perinatal/infant	X			X	X	X	X	X				
Pediatric	X				X					X		
Adult	X				X					X		
Anti-asfotase alfa IgG												X
X-Rays												
Perinatal/infant	X			X	X		X			X (dolls)	X (knees)	X
Pediatric	X				X		X			X (dolls)	X (knees)	X
Adult	X						X					X
MRI images												X
Kidney ultrasound												X
Lung study												
Perinatal/infant	X											X
Pediatric	X											X
Adult												
Growth												
Perinatal/infant	X			X	X	X	X	X (up to 4 years old)	X (over 4 years old)			
Pediatric	X							X (up to 4 years old)	X (over 4 years old)			
Motor development												
Pediatric	X								X			
Dental												
Pediatric	X									X		X
Motor function (mobility, walking, muscle strength)												
Perinatal/infant	X			X	X		X			X		
Pediatric	X				X		X			X		
Adult	X			X	X		X			X		
Pain												
Perinatal/infant	X		X (C/month for 6 months)	X								
Pediatric	X				X		X			X		
Adult	X				X		X			X		
Quality of life												
Perinatal/infant	X									X		
Pediatric	X				X					X		
Adult	X				X		X			X		
Safety of treatment												
Gastrointestinal symptoms (GER and aspiration)												
Pediatric	X				X		X			X		
Adult	X											X
Nutrition	X									X		X
Pediatric	X									X		X
Bone biopsy												
Adult												X

Source: adapted from [19]
2. Musculoskeletal complications, such as chronic pain and osteoarthropathies (including pseudo-gout attacks), should be evaluated through comprehensive clinical examinations. (Strong in favor).
3. Growth and development in pediatric patients should be tracked using anthropometry and validated neurodevelopment scales to identify short stature and developmental delays. (Strong in favor).
4. Regular dental consultations are recommended to ensure oral health. (Strong in favor.
5. Fixed or removable prostheses are recommended for pediatric patients with early tooth loss until they develop permanent teeth. (Strong in favor).
6. Implant management is recommended for adults with tooth loss. (Weak in favor).
7. Annual ophthalmology follow-up is recommended for ERT patients to identify ectopic ocular calcification. (Strong in favor).
8. Motor function evaluation through validated scales or tests is recommended, such as the 6-minute walk test for people over five years old and the AIMS or GMFM for younger children. (Strong in favor).
9. Quality of life monitoring is suggested using validated scales such as SF-36 until a specific scale for HPP is developed. (Strong in favor).
10. Routine ALP measurement is not recommended except in cases of suspected poor adherence to ERT. (Strong against).
11. In patients with good adherence to ERT but persistently low ALP levels and no clinical or radiological improvement, the presence of neutralizing antibodies should be suspected and measured if available. (Expert opinion).
12. Tracking serum calcium and phosphorus, PTH, vitamin D (25-hydroxyvitamin D), blood count, liver function (bilirubin, ALT, AST), and electrolytes is recommended. (Strong in favor).
13. Monitoring renal function through creatinine, eGFR, and the calciuria/creatinuria ratio in an isolated urine sample or 24-hour calciuria (depending on age) is recommended. (Strong in favor).
14. Diagnosis and monitoring of nephrocalcinosis through renal ultrasonography is suggested in patients with altered calcium-phosphorus metabolism. (Strong in favor).
15. Individualized radiological follow-up is suggested to identify chondrocalcinosis, deficiency in fracture consolidation, atypical femoral fractures, and pseudofractures. (Strong in favor).
16. Bilateral radiographs of the wrists and knees are recommended to evaluate the radiographic Rickets Severity Score. (Strong in favor).
17. Bone densitometry is not suggested as part of the bone metabolism follow-up study in patients with HPP. (Strong against).
18. Bone biopsy is suggested for adult HPP patients with CKD, a history of low bone mineral density, or fractures. (Expert opinion).

#### Evidence

Despite the variability of HPP presentation, strict patient follow-up is consistently highlighted in the literature [19, 26, 57]. Clinical, biochemical, and imaging tracking allow for the evaluation of the disease progress, identification of complications, ERT effectiveness and safety, and adjustment of medical treatment [26, 57].

Clinical follow-up includes respiratory function assessment, anthropometric measurements such as weight [14], height, head circumference, and growth velocity, as well as evaluations of gait pattern, pain, motor function, quality of life (measured with validated scales) [19, 26], and dental condition [26]. For pediatric patients under five years old, motor evaluation using the Gross Motor Function Measurement (GMFM), or the Alberta Infant Motor Scale (AIMS) is suggested. For patients over five years old, the 6-minute walk test (6MWT) is recommended [19, 58]. Although there are no specific quality-of-life scales for HPP patients, the significant impact of the disease on quality of life has been elucidated through surveys designed for HPP patients [59].

ALP activity should be measured only in cases where poor adherence to ERT is suspected. Biochemical monitoring includes measuring of calcium, phosphorus, PTH, vitamin D (25-hydroxyvitamin D) [53], blood count, liver function (bilirubin, ALT, AST), electrolytes, and kidney function through creatinine, eGFR, calciuria/creatinuria ratio in an isolated urine sample or 24-hour calciuria. The frequency of these tests varies depending upon age group and presentation type; likewise, the results must be interpreted according to age and sex [19]. While the measurement of anti-Asfotase alfa antibodies (ADA) has been described, this monitoring alternative remains under investigation and is not commercially available [19]. Although the measurement of PLP and PPi in plasma and PEA in urine has been mentioned, evidence has not yet shown that changes in these parameters, correlate with clinical and radiological findings [24].

Regarding imaging follow-up, X-rays are recommended in pediatric and adult patients. For perinatal or infantile HPP, chest X-rays should be taken, and for all pediatric age presentations, comparative wrists and knees radiography are needed [19]. These should be ideally analyzed using the Rickets Radiographic Severity Score and at follow-up with the Radiographic Global Impression of Change [26].

Renal ultrasonography to identify nephrocalcinosis and kidney stones has been proposed as an ERT follow-up tool with a semiannual or annual periodicity [57]. Once craniosynostosis is diagnosed, it should be monitored until complete brain formation; at least until 12 years of age [14].

A bone biopsy could be helpful in adult patients on ERT treatment for conditions such as CKD, a history of fractures, or very low bone mineral density. The usefulness of bone densitometry as a follow-up tool is still controversial [19].

## Conclusion

The recommendations in our consensus aim to facilitate early and timely diagnosis, standardize treatment, and streamline patient follow-up for individuals with hypophosphatasia. We strive to positively impact the disease burden and improve health outcomes, including patient survival and quality of life.

## Supplementary information

Supplementary Information

## Data Availability

No datasets were generated or analysed during the current study.
