# Supplementary material for: Diagnosis, treatment, and follow-up of patients with hypophosphatasia
Source: Endocrine. 2024 Dec 12;87(2):400–19. doi: 10.1007/s12020-024-04054-1 (PMC11811241; doi:10.1007/s12020-024-04054-1)
Supplement: Supplementary file 1 — Supplementary Information [file 12020_2024_4054_MOESM1_ESM.docx]

**Materials and methods supplement**

This is an expert consensus, based on the most recent and highest quality published clinical evidence. The developer group was enrolled including specialist in pediatric nephrology, pediatric endocrinology, adults endocrinology, clinical genetics, infantile orthopedics, pediatric rheumatology, epidemiology, and methodologists with experience in systematic review and expert consensus.

After an analysis of the disease and thinking in the best interest for hypophosphatasia patients, nine clinical questions were selected. A systematic review (SR) was carried out through Medline and Embase searching for published evidence that will help answering the questions. Grey literature was searched through Google Scholar, references lists, the developer group’s official website of Clinical Practice Guidelines (CPG), health technology assessment (HTA), and consensus documents. The search strategy details are in Annex 1. and documents in Spanish or English with recommendations published between September 2012 and March 2024 were included.

Annex 1. Search strategy

| **Source** | **Search strategy** | **Results** |
| --- | --- | --- |
| Pubmed | ((((Hypophosphatasia[Title/Abstract]) OR (ALPL[Title/Abstract])) OR (TNSALP[Title/Abstract])) OR (Hipofosfatasia[Title/Abstract]) AND (y_10[Filter])) AND ((((diagnosis) OR (Therapeutics)) OR (enzyme replacement therapy)) OR (follow) AND (y_10[Filter])) | 602 |
| Embase | (hypophosphatasia:ti,ab OR 'alpl gene'/exp OR tnsalp:ti,ab) AND ('diagnosis'/exp OR 'bacteriologic diagnosis' OR 'diagnosis' OR 'diagnostic screening' OR 'diagnostic screening programs' OR 'diagnostic sign' OR 'diagnostic tool' OR 'diagnostics' OR 'disease diagnosis' OR 'medical diagnosis' OR 'physical diagnosis' OR 'therapy'/exp OR 'combination therapy' OR 'disease therapy' OR 'disease treatment' OR 'diseases treatment' OR 'disorder treatment' OR 'disorders treatment' OR 'efficacy, therapeutic' OR 'illness treatment' OR 'medical therapy' OR 'medical treatment' OR 'multiple therapy' OR 'polytherapy' OR 'somatotherapy' OR 'therapeutic action' OR 'therapeutic efficacy' OR 'therapeutic trial' OR 'therapeutic trials' OR 'therapeutics' OR 'therapy' OR 'therapy, medical' OR 'treatment effectiveness' OR 'treatment efficacy' OR 'treatment, medical' OR 'follow up'/exp OR 'follow up' OR 'follow up study' OR 'follow-up studies' OR 'followup' OR 'lost to follow up' OR 'lost to follow-up') AND [2012-2022]/py  Fecha de búsqueda: 25 de septiembre de 2022 | 902 |
| Google Scholar | Hypophosphatasia AND guidelines | 5 |
| NICE | Hypophosphatasia | 0 |

The information was independently screened and selected by two reviewers (clinical-methodologists) (Annex 2). The quality of the evidence was evaluated according to the document type: AGREE II for Clinical Practice Guidelines (CPGs), ROBIS for systematic reviews (SR), and the Joanna Briggs Institute approach for primary studies.

A modified Delphi Consensus was made. The first step consisted in online synchronous sessions for evidence analysis and the developer group proposed recommendations based on evidence and expertise. Secondly, external clinical experts analyzed the recommendations in online sessions, and voted for each recommendation (An agreement threshold of at least 80% was defined for each recommendation to be included in the consensus). Two discussion and vote rounds were carried out, and agreement was achieved on all recommendations.

Annex 2. Evidence included process

Included

n = 61

Number of references screened

n = 1127

n = 884

Excluded due to: Study type and not within the publication period (September 2012 to March 2024)

Number of full-text articles evaluated for eligibility

n = 243

n= 396 duplicates

n=182

Excluded due to lack of clinical relevance, and poor level and quality of evidence

Number of electronic database references

n =1504

Number of references from other sources

n = 19

Source: Own elaboration.

The recommendations were graded by the clinical experts based on intensity (strong or weak/conditional) and direction (in favor or against).

- **Strong in favor:** Clinical experts believe that the practice promoted in the recommendation offers more benefits than risks for most patients and should be implemented in daily practice.
- **Weak in favor:** Clinical experts believe that patients could benefit from the practice promoted in the recommendation, although certain patient conditions, contexts, or new evidence could alter the recommendation.

For the categories **Strong Against** or **Weak Against**: the practice is unadvisable because it could pose greater risks than benefits to the patient, or the evidence is inconclusive.

In the absence of supporting evidence, recommendations or suggestions were not graded and were classified as **Expert opinions**.

Overall, the quality of the evidence was low. Study design (mainly case series, case reports or reviews) was the principal methodological limitation in the reviewed literature. Some documents presented a post-hoc analysis and the only available clinical practice guideline has limitations in methodology and editorial independence.

It is very important to acknowledge that HPP is a rare disease, therefore, studies with a larger number of subjects are nearly impossible. In despite of these challenges, the recommendations in this document are based on the best available evidence.
